# Host genetics and population structure effects on parasitic disease

**DOI:** 10.1098/rstb.2011.0296

**Published:** 2012-03-19

**Authors:** Sarah Williams-Blangero, Charles D. Criscione, John L. VandeBerg, Rodrigo Correa-Oliveira, Kimberly D. Williams, Janardan Subedi, Jack W. Kent, Jeff Williams, Satish Kumar, John Blangero

**Affiliations:** 1 Department of Genetics, Texas Biomedical Research Institute, PO Box 760549, San Antonio, TX 78245, USA; 2 Department of Biology, Texas A&M University, 3258 TAMU, College Station, TX 77843, USA; 3 Southwest National Primate Research Center, PO Box 760549, San Antonio, TX 78245, USA; 4 Centro de Pesquisas, René Rachou, FIOCRUZ Minas, Av. Augusto de Lima 1715, Belo Horizonte, Minas Gerais, Brazil; 5 Department of Anthropology, Temple University, 210 Gladfelter Hall, 1115 Polett Walk, Philadelphia, PA 19122, USA; 6 Department of Sociology and Gerontology, Miami University, Upham 358, Oxford, OH 45056, USA

**Keywords:** population structure, genetics of infectious disease susceptibility, intestinal worms, Chagas disease

## Abstract

Host genetic factors exert significant influences on differential susceptibility to many infectious diseases. In addition, population structure of both host and parasite may influence disease distribution patterns. In this study, we assess the effects of population structure on infectious disease in two populations in which host genetic factors influencing susceptibility to parasitic disease have been extensively studied. The first population is the Jirel population of eastern Nepal that has been the subject of research on the determinants of differential susceptibility to soil-transmitted helminth infections. The second group is a Brazilian population residing in an area endemic for *Trypanosoma cruzi* infection that has been assessed for genetic influences on differential disease progression in Chagas disease. For measures of *Ascaris* worm burden, within-population host genetic effects are generally more important than host population structure factors in determining patterns of infectious disease. No significant influences of population structure on measures associated with progression of cardiac disease in individuals who were seropositive for *T. cruzi* infection were found.

## 1. Introduction

### (a) Genetic determinants of infectious disease susceptibility

Host genetic factors exert significant influences on differential susceptibility to many infectious diseases [1–3]. Our group has been working on the genetic determinants of susceptibility to parasitic disease since the early 1990s [4,5]. This work has resulted in the genetic characterization of susceptibility to infection both in intestinal worm infections and Chagas disease, and in the mapping of quantitative trait loci influencing soil-transmitted helminthiases [4–9]. The discussion meeting on ‘Human evolution, migration, and history as revealed by genetics, immunity, and infection’ provided the impetus to explore the potential influence of population structure on patterns of infectious disease in two populations which our group had already studied.

### (b) The Jiri Helminth Project

The Jiri Helminth Project was established in 1995 with the goal of determining the genetic components of susceptibility to helminthic infection in an endemic population [5,6]. The study was based in the Jiri region of Dolakha district in the eastern hills of Nepal, and focused on the Jirel ethnic group. Some ethnohistorical accounts and population genetic studies have suggested that this ethnic group is a hybrid population derived from Sunwars and Sherpas [10,11]. Members of the Jirel population were sampled from across seven villages in the Jiri region. All sampled members of the population belong to a single highly complex pedigree, which is one of the largest and statistically most powerful human pedigrees available for genetic analysis. The pedigree of the population was reconstructed using the approach outlined by Williams-Blangero & Blangero [12].

Phenotypic information was determined for members of the population in the following manner as is described by Williams-Blangero *et al*. [5]. First, a meeting was held at the village level in each of the villages to explain the purpose of the study and encourage participation. Following these general meetings, recruitment of study participants was carried out on a household basis. Individuals aged 3 years and older were recruited following a protocol approved by the University of Texas Health Science Center at San Antonio Institutional Review Board and the Nepal Health Research Council. Following enrolment, there was a clinic visit for collection of blood samples for extraction of DNA, and all individuals were given a dose of albendazole, an effective anthelmintic drug. Individuals were provided with collection containers to take home, and all stools passed over the 4 days following treatment were collected. Faecal samples were filtered to obtain worm counts for each individual host, and worms were weighed to determine total worm weight for each individual host [5].

The Jirel study participants have been genetically characterized as part of a long-term genome-wide scan study [6–8], and a portion of the population has been characterized using the Illumina 660 BeadChip. Genome-wide single nucleotide polymorphism (SNP) data are available for a total of 819 individuals. The roundworms collected from the Jirel participants have been characterized for 23 microsatellites each, and genetic data are available for a total of 1094 worms collected from 320 people belonging to a total of 165 households [13,14].

### (c) The Posse Family study

The Posse Family study was initiated in 1996 to characterize genes that influence susceptibility to infection with the parasitic organism *Trypanosoma cruzi* and subsequent cardiac disease progression in American trypanosomiasis or Chagas disease. The rural Posse region of Goiás, Brazil is endemic for Chagas disease with approximately 50 per cent of the adult population being seropositive for *T. cruzi* infection [9]. *T. cruzi* is transmitted to humans by triatomid or kissing bugs [15]. The bugs live in the mud walls and thatched roofs of houses in endemic areas, and descend at night to feed on the human host. Chagas disease results from long-term infection with *T. cruzi* [15].

Sampling for our study of Chagas disease was performed on a house-to-house basis in the villages of the Posse region. We collected blood samples to determine seropositivity for *T. cruzi* infection and for genetic assessments. Additionally, we collected electrocardiograms (ECGs) from each sampled individual in order to measure aspects of cardiac disease progression in *T. cruzi*-infected individuals [9,16]. Pedigrees were reconstructed as described by Williams-Blangero & Blangero [12]. Although primarily of Hispanic European descent, this population is expected to be substantially more admixed than the Jirel population. Both Amerindian and African admixture are prominent in much of Brazil.

### (d) Population structure and infectious disease susceptibility

Our work on Chagas disease and intestinal helminth infections has resulted in the mapping of genes that influence helminthic infection and in the characterization of genetic components that influence Chagas disease. However, these are complex diseases with multi-factorial determinations and the question of whether or not underlying population structure influences infectious disease patterns is an intriguing one. In addition, parasite genes and population structuring of the parasites themselves may be important determinants of disease patterns in the host population [13,14].

Populations are substructured along many lines, including family structure which we study extensively as part of our genetic studies. However, they are also substructured at higher levels including by geographical distance, by differential admixture and by genetic drift among subpopulations.

## 2. Material and methods

### (a) Host and worm genetic effects on host *Ascaris* burden

We first wanted to determine if both host genes and worm genes are important for determining the distribution of *Ascaris* worm burden in the Jirel population. For this analysis, we used data available for 320 individuals (including 141 males and 179 females ranging in age from 3 to 79 years). The 1094 *Ascaris* worms collected from these individuals were characterized for a total of 23 genetic markers [13,14].

Using variance decomposition theory, we derived a model that allowed the infection-related phenotypic variance to be decomposed into components that are owing to host genetic factors, genetic variation among the worms and random environmental factors. For *n* host individuals, the *n* × *n* phenotypic covariance matrix (***Ω***) is given by the following variance component model:
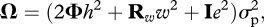
where 2***Φ*** is twice the kinship matrix (i.e. the coefficient of relationship matrix) among individuals which is derived from the host pedigree structure, **R***_w_* represents the estimated coefficient of relationship [17] between parasites across hosts (and averaged within host) as measured (see Criscione *et al*. [14]) from highly polymorphic short tandem repeat polymorphism in the *Ascaris* worms collected from the Jirel human host population, and **I** is the identity matrix. The phenotypic variance of the focal trait is given by 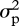 while the relative variance components associated with each of the structuring matrices are the additive genetic heritability in the hosts (*h*^2^), the additive genetic heritability in the worms (*w*^2^) and the proportion of phenotypic variance owing to random environmental factors (*e*^2^ = 1 − *h*^2^ − *w*^2^).

Using a standard maximum-likelihood variance estimation method as implemented in the statistical genetics computer package, SOLAR [18], we can estimate the parameters of this model along with any mean effect parameters (such as covariate effects). In order to estimate this model, we assume multivariate normality, which is obligately violated for traits such as worm counts. Therefore, we performed direct inverse Gaussian transformations on all phenotypes prior to analysis. Even after such transformation, these traits are not exactly normal. However, prior analyses have shown that as long as kurtosis is not excessive, the maximum-likelihood analyses provide valid results [19].

Using this approach, we tested whether nested models that eliminate specific variance components adequately captured the variation accounted for in the most general model (shown above). Likelihood ratio tests (LRTs) were formed as twice the difference in ln likelihoods. Because of boundary conditions (variances must be greater than or equal to zero), the resultant test statistics are distributed as mixtures of chi-squared distributions [20]. For example, when comparing a model that includes both host and worm genetic variance components with one only including host genetics (i.e. *w*^2^ = 0), the LRT is distributed as 50 : 50 mixture of a point mass at zero and a chi-squared distribution with 1 d.f. When comparing the more general model with a random environmental model in which both *h*^2^ and *w*^2^ are constrained to zero, the resulting LRT is distributed as 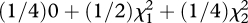.

All worm burden traits were normalized using an inverse Gaussian transformation prior to analysis. Covariates for worm burden as assessed by *Ascaris* total count included sex, age and number of days of sampling post-albendazole. Only age was significant (showing a decrease in total worm count with age, *p* = 0.0160). Covariates for worm burden as measured by total *Ascaris* weight included sex, age, number of days of sampling post-albendazole and sex ratio of worms. Age (decrease in total worm weight with age, *p* = 0.0541), number of sampling days (increase in total worm weight with number of days, *p* = 0.0207), and sex ratio (increase in total worm weight with higher proportion of female worms, *p* = 2.6 × 10^−9^) were significant.

### (b) Host genetic and spatial effects on host *Ascaris* burden

Because our sample size of genotyped worms by host was relatively small, we also decided to assess potential spatial effects influencing host worm burden. The inclusion of a random spatial process may also allow inference on worm population structure effects, such as inference of isolation by distance or spatial autocorrelation effects on worm kinship [14]. For this analysis, we used data on 1108 individuals (544 males and 564 females ranging in age from 3 to 85 years). The covariates considered in the analysis included sex, age and number of sampling days for *Ascaris* counts, and sex, age, number of days sampling and for *Ascaris* weight, sex ratio of the worms.

We employed a standard exponential decay model to parametrize the expected correlations between hosts as a function of geographical distance. Because host genetic factors were explicitly accounted for by the pedigree information, any resulting spatial component may represent either: (i) a geographical component of exposure patterns owing to local environmental conditions such as temperature or moisture or (ii) a component resulting from worm population differentiation resulting from an isolation by distance process. In order to account for such a potential spatial process, we amend our previous variance component models as follows:
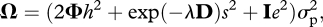
where **D** is a matrix of geographical distances (in kilometres) between individuals as assessed from geographical coordinates of the houses that they live in, λ is the exponential decay parameter and *s*^2^ is a new relative variance component that measures the relative proportion of variance accounted for by the random spatial process. The term exp(−λ**D)** generates a valid spatially decaying positive correlation matrix that structures the spatial variance component. As λ → ∞, this model becomes a standard shared household model in which individuals within a household exhibit a correlation of 1, while across households the resulting correlation is 0. This model can also be expanded to allow for host genetic × spatial interactions.

### (c) Effect of Jirel population structure on *Ascaris* worm burden

While our models specifically included host within-population genetic variation, there is also the possibility that some genetic between-group variation influences worm burden measures. Such between-group variation may have resulted from admixture or gene flow with other ethnic populations or be the result of microevolutionary processes that lead to differentiation between Jirel villages. Given the clan-structured nature of Jirel villages and mate choice [21], we may expect some systematic between-group differentiation.

In order to test for the potential effects of Jirel population structure, we performed principal component analysis (PCA) on SNP genotypes obtained from Illumina 660 BeadChip. SNP data were available for 819 individuals (404 males, 415 females). A PCA panel of SNPs was selected from a full panel of haplotype-tagging SNPs based on the following criteria: minimum 1 kb spacing across the autosomal genome; minor allele frequency greater than or equal to 10 per cent; and pair-wise linkage disequilibrium ≤ |*ρ*| = 0.15 within a sliding 1 Mb window. For the Jirel cohort, genotypes for 26 272 polymorphic SNPs (out of 561 340 total) were used in 200 founders and other unrelated persons selected from 916 genotyped subjects. The principal components (PCs) were derived only from unrelated individuals in order to avoid detecting structure induced by close biological relationships. SNPs were scored for dosage (zero to two copies) of the minor allele of each SNP. PCA was performed using the prcomp procedure in R (http://www.r-project.org), and scores were predicted for the remaining related individuals based on their genotypes. The resulting PCs should only reflect population genetic structure such as systematic gene flow or local admixture or intra-population differentiation such as between-village differences owing to drift and migration patterns. In the unrelated Jirel individuals, the cumulative proportion of total genotypic variance explained by the first four PCs was 4.2 per cent, consistent with the very flat distribution of variance across PCs. This effectively uniform distribution suggests that there is relatively little structure in the Jirel population and the structure that is observed probably reflects local admixture with relatively closely related populations and/or Jirel microdifferentiation owing to non-random migration among villages [22].

In order to test whether Jirel population structure influences worm burden, we performed fixed-effect tests on worm burden using a linear regression model in which SNP-derived PCs were employed as covariates. Because of the relative lack of evidence for embedded genetic structure in the Jirel population (as observed by the small eigenvalues obtained during the PC analysis), we limited formal testing of population structure to the first two PCs. As with our previous analyses, we also included covariates such as sex, age, number of days sampled post-albendazole treatment and sex ratio of worms (for *Ascaris* weight only). These analyses were embedded in a variance component framework in which we explicitly allowed for host additive genetic effects. This allows for adequate handling of the non-independence among individuals and also eliminates the potential for the test of the PCs to be biased owing to their reflection of close biological kinship. A likelihood ratio test was calculated for each tested PC by comparing a model in which the regression coefficient on the PC was estimated with one in which the regression coefficient was constrained to zero. This provided a LRT that is distributed as a chi-square with a single degree of freedom.

### (d) Effect of Posse population structure on Chagas disease-related traits

Similar to our above analysis of the Jirel population, we performed PCA on high-density SNP data on samples obtained from the Posse population. These samples were limited to *T. cruzi*-infected individuals. Using the same SNP selection process as described above, we selected 25 740 out of 1.14 M total SNPs (obtained using the Illumina HumanOmni1-Quad chip). The currently genotyped sample is substantially smaller than our Jirel example. For this smaller cohort, we identified 49 unrelated individuals (out of 559 total *T. cruzi*-infected individuals with complete data) to perform the PCA. From these results, we used the estimated PC coefficients to project PCs for the rest of the related sample. As expected, there was substantially greater genetic population structure in the Posse sample relative to that observed in the Jirels, with the first four PCs of the SNP variation cumulatively explaining 13.4 per cent of total variance (i.e. greater than three times that observed in the Jirels). This greater evidence for systematic population structure is presumably the result of larger scale admixture in the Brazilian population.

As with our analyses of the effects of population structure on worm burden in Nepal and given the greater *a priori* evidence for population structure in the Posse population, we performed fixed effect-based effects on the first four PCs allowing for residual non-independence among related individuals owing to additive genetic effects. For each phenotype, we also simultaneously allowed for covariate effects including sex, age, sex × age, age^2^, sex × age^2^ and height in all analyses. The sample consists of 297 females and 262 males with a mean age of 51.5 years. Besides quantitative measures from ECGs (see Williams-Blangero *et al*. [16] for a description), we examined two dichotomous indicators: right bundle branch block with a prevalence of 16.8 per cent in this sample and any discrete ECG abnormality (prevalence = 46.7%).

## 3. Results

### (a) Host and worm genetic effects on host *Ascaris* burden

Table 1 shows the results of our initial variance component analysis assessing host and worm genetic effects on host *Ascaris* burden. For *Ascaris* total count, the model which included only host genes fits as well as the most general model which included both host genetic and worm genetic effects. Thus, the host genetic model represents a parsimonious model that fits as well as a more general model that included both host and worm genetic factors. Additive genetic host factors account for approximately 54 per cent of the total variation in worm counts. Models that only included worm genetic factors or no genetic factors were both rejected as being significantly different from the general model.

**Table 1.** Results of joint analysis of host and worm genetic effects on *Ascaris* burden phenotypes.

trait	model	*h*^2^	*w*^2^	*p*-value
*Ascaris* total count				
	host genes + worm genes	0.539	0	—
	host genes only	0.539	0	0.5000
	worm genes only	0	0.515	0.0090
	random environment	0	0	6.8 × 10^−9^
*Ascaris* total weight				
	host genes + worm genes	0.166	0.218	—
	host genes only	0.369	0	0.2748
	worm genes only	0	0.384	0.3158
	random environment	0	0	2.3 × 10^−4^

One might expect host genetic effects to be the primary influence on total worm count because each worm represents an independent infection. There would be strong selection for infectivity in the worms because infection must occur in order for the worm to reproduce. Hence, worm counts are a function of the survival of individual worms and therefore reflect fitness-related traits that would be expected to have low levels of genetic variation.

A characteristic that is more likely to be influenced by worm genetic factors is total worm weight in the host, because weight variation in the common host environment is a characteristic associated with the worms themselves. For host worm burden as measured by *Ascaris* weight, models allowing for only worm genetic factors or only host genetic factors could not be rejected. However, the worm genetics only model fits slightly better than the host genetics model. The environmental model could be rejected. Thus, models with either host or worm genetic factors alone fit these data. However, in the general model in which both host and worm genetic factors were fit, these parameters seemed somewhat confounded, exhibiting an error correlation of −0.953. Examination of the structure of the data revealed some confounding between host and worm coefficient of relationships, indicating that worm genetic effects and host genetic effects are highly correlated.

### (b) Host genetic and spatial effects on host *Ascaris* burden

Table 2 shows the results of the variance component analysis using the combined host genetic/spatial process model. There were 1108 individuals in this analysis including 544 males and 564 females ranging in age from 3 to 85 (mean age = 26.1 years). As with the prior analysis, covariate effects were simultaneously estimated.

**Table 2.** Results of joint analysis of host genetic effects and a random spatial component on *Ascaris* burden phenotypes.

trait	model	*h*^2^	*s*^2^	*λ*	*p*-value
*Ascaris* total count					
	host genes + spatial	0.272	0.096	3.92	—
	host genes only	0.397	0	∞	1.8 × 10^−4^
	spatial only	0	0.126	92.29	0.0292
	random environment	0	0	∞	1.6 × 10^−22^
*Ascaris* total weight					
	host genes + spatial	0.151	0.095	6.791	—
	host genes only	0.369	0	∞	2.0 × 10^−4^
	spatial only	0	0.153	69.68	0.0811
	random environment	0	0	∞	4.2 × 10^−12^

For total *Ascaris* worm count, these analyses show that both a host genetic factor and a random spatial component are required to best fit the data. All nested models are rejected when compared with the more general model in which all three variance components are estimated. The estimate of the exponential decay parameter in the general model was 3.92 which suggests that the expected spatially varying correlation is halved by a distance of 0.177 km. In the general model, host genetics accounts for approximately 27.2 per cent (±9.6%) of the total variation in *Ascaris* worm count, whereas spatial factors account for an additional 9.6 per cent (±4.1%). Thus, host genetic factors appear to be the single largest determinant of phenotypic variation. The observed spatial component may reflect the worm genetic component but this still remains to be resolved.

For worm burden as measured by total *Ascaris* weight, the data suggest that a spatial process alone can account for the observed variation. In fact, the estimated decay parameter for the spatial process only model is very high (69.68), indicating very rapid decay and suggesting that the model has reduced to a basic household sharing model where correlations between individuals are only observed within household. However, such a model also is confounded by close biological relationships within households and therefore captures some host genetics along with local exposure or extremely rapidly decaying worm population structure.

### (c) Effect of Jirel population structure on host *Ascaris* worm burden

We examined the population structure of the Jirels agnostically using high-density SNP information. PCs are routinely used to remove confounding owing to latent population structure in genome-wide association studies [23]. Here, however, we used the PCs as indicators of between-group genetic variation, and assessed whether or not the PCs of SNP covariation, which represent systematic axes of population structure, correlate with our measures of infection.

Our previous work in anthropological genetics has demonstrated that there is structuring in the Jirel population [10,11,21,22]. As noted earlier, the population is a hybrid population derived from Sunwars and Sherpas approximately 11 generations ago. We have demonstrated that there is genetic differentiation both between villages and between clans within the Jirel population.

In order to test whether Jirel population structure influences worm burden, we performed fixed effect tests on worm burden using a linear regression model in which the SNP-derived PCs were employed as covariates. Because of the relative lack of evidence for embedded genetic structure in the Jirel population as observed by the small eigenvalues obtained during the PCA, we limited formal testing of population structure to the first two PCs. As with our previous analyses, we included covariates such as sex, age, number of days sampled after treatment, and for the worm weight phenotype, within-host sex ratio of the worms was also considered as a covariate. These analyses were embedded in a variance component framework in which we explicitly allowed for host genetic effects.

The cumulative proportion of total genotypic variance explained by the first four PCs was 4.2 per cent, consistent with the very flat distribution of variance across PCs. Despite this relatively slight stratification, both focal host *Ascaris* burden traits showed some evidence of being influenced by Jirel population structure. Host *Ascaris* total count was significantly influenced by the first PC (*p* = 0.01592). It accounted for about 1.1 per cent of the total phenotypic variation in worm count. This estimate probably estimates latent between-group variation that is indexed by PC1. Given that the Jirels have been speculated to be an admixed population with genetic input from both Sherpas and Sunwars, admixture may be the source of this variation. This population structure-induced variance can be contrasted with the additive genetic heritability estimated simultaneously which was estimated to account for 47.4 per cent of the total phenotypic variation in worm count. Thus, genetic factors including both between- and within-group variation appear to be important influences on total worm burden. Similarly, host worm burden as assessed by total *Ascaris* weight also showed a significant relationship with the first PC (0.0128). Again, approximately 1.1 per cent of the phenotypic variation in *Ascaris* weight could be attributed to the population structure-induced genetic variation. Overall, these analyses suggest that some of the observed phenotypic variation in worm burden can be ascribed to underlying Jirel genetic structure.

These analyses then suggest that some of the observed phenotypic variation in worm burden can be ascribed to underlying genetic structure in the Jirel population reflective of some type of latent between-group variation. However, our results overall suggest that host genetic factors are more important than population structure factors in determining patterns of helminthic infection.

### (d) Effect of Posse population structure on Chagas disease-related traits

Table 3 shows the results of our analyses of Chagas disease-related traits in the Posse population and population structure as determined from the PCs of high-density SNP data. All traits were significantly heritable. Unlike our analysis of worm burden, we observed very little evidence for the effects of Posse population structure on our cardiac disease-related phenotypes. We observed nominally significant evidence for population structure effects only for diastolic blood pressure (for PC4, *p* = 0.043) and for the ECG-derived parameter PR interval (for PC2, *p* = 0.044). Given the larger number of phenotypes and the number of PCs, these marginal results would fail to achieve experiment-wide significance. Thus, even though there seems to be substantially more structure in the Brazilian sample, it appears that the underlying genetic structure variation within the Posse population is not important for the infectious disease traits under consideration.

**Table 3.** Effects of population structure on Chagas disease-related phenotypes.

trait	*p*-value PC1	*p*-value PC2	*p*-value PC3	*p*-value PC4
diastolic blood pressure	0.1128	0.1474	0.3218	0.0431
systolic blood pressure	0.7078	0.7645	0.3504	0.0521
QRS interval	0.6387	0.5252	0.9191	0.2017
PR interval	0.2080	0.0438	0.7936	0.3571
QT interval	0.9249	0.8539	0.5878	0.1763
ventricular rate	0.8386	0.9721	0.1133	0.3672
abnormal ECG	0.6987	0.9643	0.2548	0.7386
right bundle branch block	0.5054	0.8779	0.7714	0.2365

## 4. Discussion

The results of the analyses presented here demonstrate the value of considering population structure effects on variation in parasitic disease burden. Evidence for population structure effects may be informative for understanding the importance of genetic variation in the parasite itself. However, population structure effects on infectious disease burden are unpredictable. Even in two populations with known structuring, evidence for population structure effects on patterns of parasitic disease is limited. It is important to consider simultaneously the effects of population structure and of host and parasite variation in determining disease patters. Our results suggest that within-population host genetic factors are generally more important than host population structure factors in determining patterns of infectious disease.

We primarily view the observed population structure effects as reflective of underlying between-group genetic variation and hence would not expect between-group genetic variation to be a larger determinant than within-group genetic variation. In classical population genetic models, between-group genetic variation is expected to be proportional to within-group genetic variance, with the relationship being a scalar function of Wright's *F*_ST_ between the appropriate ancestral or current groups involved in gene flow. Even for major ethnic group differentiation, this parameter rarely is as large as 15 per cent of the within-group variation [24]. In the Jirels, observed population structure effects are probably owing to the residual effects of differential admixture with relatively closely related ancestral populations including Sherpas and Sunwars. Additionally, the relative endogamy of Jirel villages [11,25] and the non-randomness of clan-structured migration [21] lead to a second type of lesser microdifferentiation generating local between-group genetic differences. We appear to have observed some of this between-group genetic variation as relevant for our helminthic infection-related phenotypes.

In our Brazil study population, we fully expected to see larger admixture-related between-group genetic effects for our disease-related variables. The Brazilian population exhibits substantial admixture between local populations of very diverse ancestry including major African and Amerindian components against a European ancestral background. Our lack of finding major between-group genetic variation in Chagas disease-related phenotypes may suggest lack of differences in susceptibility/response to this infection across ethnic groups. However, there is still strong evidence for major within-group genetic variation influencing these phenotypes [9].

In this study, we have focused on genetic factors in aggregate, which is sometimes termed the polygenic component of phenotypic variation. Our variance component models incorporate aggregate additive genetic effects in both host and parasite populations (although sometimes very indirectly for the parasite population). However, the central goal of our ongoing studies of these two isolated human populations is the identification of specific host genes lying in the causal pathway of susceptibility to infection or the phenotypic response to infection. In fact, the high-density genetic markers that we use in this study were originally generated specifically to perform quantitative trait locus (QTL) localization for host genes influencing infection. Their primary use has been to track segments of DNA that are passed identical-by-descent across the generations in order to co-localize them with phenotypic variation. We have previously localized major QTLs influencing helminthic infections in the very large Jirel pedigree [6,7], including our continuing efforts to identify a major causal gene influencing *Ascaris* infection on chromosome 1. In general, we expect the underlying genes whose sequence variation leads to differential infection susceptibility or response to play major roles in immunological response to pathogens. However, we are still in the early days of causal gene identification although we are confident that the growing availability of inexpensive high-throughput next-generation sequencing will speed the pace of such discovery. Regardless of our long-term aims for uncovering the specific genes involved in differential response to infection, in this study, we focused on a secondary use of high-density genetic marker data that is generally employed to correct for the potentially adverse effects of latent population structure in gene localization studies. Thus, what is typically a nuisance factor in a study of within-group genetic variation becomes the focal factor in a study of between-group genetic variance.
